# Physical Examination-Indicated Cerclage in Singleton and Twin Pregnancies and Risk Factors for Predicting Preterm Birth < 28 Weeks

**DOI:** 10.3390/jpm14010038

**Published:** 2023-12-28

**Authors:** Ji-Eun Song, Suyeon Park, Jiwon Ryu

**Affiliations:** 1 Department of Obstetrics and Gynecology, Hallym University College of Medicine, Kangnam Sacred Heart Hospital, Seoul 07441, Republic of Korea; jwryu@hallym.or.kr; 2 Department of Obstetrics and Gynecology, Inha University College of Medicine, Inha University Hospital, Incheon 22332, Republic of Korea; sypark0438@inha.ac.kr

**Keywords:** twin pregnancy, singleton pregnancy, physical examination-indicated cerclage, preterm birth, predictor, cervical length

## Abstract

We compare the outcomes of physical examination-indicated cerclage (PEIC) between singleton and twin pregnancies and analyze predictive factors for preterm birth < 28 weeks of gestation. Patients who underwent PEIC at our center were reviewed. We compared perinatal outcomes between singleton and twin pregnancies. The primary outcome was delivery before 28 weeks of gestation. Also, we analyzed perioperative clinical, laboratory, and sonographic findings to determine the risk factors for predicting preterm birth < 28 weeks. The rate of preterm birth < 28 weeks was not significantly different. Also, neonatal outcomes were not different. Also, we compared the outcomes according to GA (gestational age) at delivery before (Group A) or after (Group B) 28 weeks, which is the primary outcome. In perioperative findings, group A was likely to have more advanced cervical dilatation, bulging membranes into the vagina, positive fFN or IGFBP-1, and shorter postoperative CL (cervical length) than group B. Also, positive fFN or IGFBP-1 and postoperative CL < 21.6 mm were independently associated with a higher risk of preterm birth < 28 weeks. These findings provide the effectiveness of PEIC with twin pregnancy as well as singleton pregnancy and helpful predictive methods that might effectively identify women at high risk of preterm birth < 28 weeks following PEIC.

## 1. Introduction

Preterm birth (PTB), defined as delivery before 37 weeks of gestation, significantly contributes to perinatal mortality and neurodevelopmental impairment [1,2]. Neonatal survival rate and long-term complications improve with prolonged gestational age (GA) [2]. The survival rate of neonates born at 22 weeks of gestation is approximately 6%; however, neonates born at 28 weeks have a survival rate of >90% [1,3,4]. The twin pregnancy rate has recently increased due to advances in maternal age and assisted reproductive techniques (ART) [5]. Despite recent improvements in perinatal management, the risk of PTB in twin gestations is much higher than in singleton gestations [2,5]. PTB is 12 times higher in the twin gestation group than in the singleton group [6]. Approximately 60% of twin gestations are delivered before 37 weeks of gestation; conversely, approximately 12% of singletons are born PTB [5,6]. Physical exam-indicated cerclage (PEIC) is an intervention advocated to improve delivery outcomes in patients with painless cervical dilatation and visible or prolapsed membranes. The proposed mechanisms of PEIC that might improve pregnancy outcomes include reinforcing cervical integrity and preventing further inflammatory reactions by keeping the cervix closed. Although numerous studies have demonstrated the effectiveness of PEIC in singleton pregnancies, there is a paucity of data on the efficacy of PEIC in twin gestations. Few studies have evaluated the clinical utility of PEIC in preventing PTB in twin pregnancies. In twin gestations, PEIC has been reported to significantly prolong pregnancy by a mean difference of 6.76 weeks and reduce PTB at GA < 28 weeks compared to expectant management (31.6% vs. 89.45%; adjusted odd ratio [OR]: 0.05; 95% confidence interval [CI]: 0.01–0.2) [6]. Some investigators have compared PEIC in twin and singleton pregnancies and reported favorable pregnancy and perinatal outcomes with PEIC in twin gestations [6]. A recent systematic review and meta-analysis showed that PEIC may benefit twin pregnancies with a dilated cervix of >10 mm [7]. PEIC in twin gestations significantly prolongs pregnancy by an average difference of 6.78 weeks, reducing the risk of PTB at <34 weeks, <32 weeks, and <28 weeks of gestation [7].

Although few studies have demonstrated the benefits of PEIC in twin pregnancies, twin pregnant women with cerclage are still at the highest risk of PTB. Most studies on PEIC in twin pregnancies have focused on clinical characteristics such as preoperative cervical status, GA at delivery, and neonatal outcomes compared with PEIC in singleton pregnancies. However, no studies have reported detailed comparisons of clinical, laboratory, and ultrasound assessments between twin and singleton pregnancies with PEIC and detailed pregnancy outcomes. Furthermore, we investigated predictive screening modalities that may identify women at a high risk of PTB before 28 weeks of gestation.

## 2. Materials and Methods

We conducted a retrospective cohort study of patients who underwent PEIC and delivered at our center between January 2014 and December 2022. This study was approved by the institutional review board (approval no. 2023-06-024), and the work has been reported in line with the STROCSS criteria [8]. The cohort study included singleton or twin pregnancies between 16 0/7 and 24 6/7 weeks of gestation with painless cervical dilatation (≥1 cm) and visible or prolapsed membranes. Dichorionic diamniotic twin pregnancies were also included in the study. Monochorionic monoamniotic or monochorionic monoamniotic twin gestations were excluded because of significant differences in general management and risk of fetal or placental abnormalities. Speculum examination confirmed cervical dilatation, with visible membranes in the external os or prolapsed membranes in the vagina. Exclusion criteria included a short cervix on vaginal ultrasonography, excessive vaginal bleeding, threatened preterm labor (PTL) or PTL, fetal congenital malformations or aneuploidy, prolapsed membranes that already had prior cerclage during the same pregnancy, or maternal pre-existing medical conditions that influence the prognosis of pregnancy.

The techniques used for PEIC have been previously described [9]. Briefly, a McDonald suture was placed using Mersilene tape (Mersilene RS22, Ethicon LCC, California, USA), and the knot was tied at the 12 o’clock position under general anesthesia. Amnioreduction was performed selectively in patients with profoundly prolapsed membranes in the vagina to reduce the pressure on the membranes for ease of PEIC placement. Perioperative adjuvant treatments such as tocolytics, antibiotics, or vaginal progesterone were administered to all patients who underwent PEIC. Because cervical dilatation might be the risk of inflammation or infection, all patients were administered prophylactic antibiotics such as cephalosporin, metronidazole, and azithromycin for at least 7 days. The combination of ceftriaxone, clarithromycin, and metronidazole might be beneficial for treating and preventing intra-amniotic inflammation/infection in patients with preterm PROM (PPROM) [10]. PEIC is a very high risk of PPROM. So, we used a similar antibiotic regimen. After 1 day post-PEIC, transvaginal ultrasonography was routinely performed to measure postoperative cervical length (CL). CL and cervical funneling were measured according to standard criteria. The PEIC tie was removed at 37 weeks of gestation or earlier when clinically indicated, and all patients were delivered at the same institution. Electronic medical records were reviewed for maternal demographics, obstetric history, antepartum course, timing and indication for delivery, pregnancy and neonatal outcomes, postoperative sonographic parameters, and perioperative laboratory findings.

First, we compared the demographic characteristics, perioperative clinical, laboratory, sonographic findings, and pregnancy and neonatal outcomes between twin and singleton gestations who underwent PEIC. The primary outcome was PTB before 28 weeks of gestation. Secondary outcomes included GA at delivery, PTB before GA 32 weeks and GA 34 weeks, cerclage to delivery latency, perioperative clinical, laboratory, and sonographic details at the time of PEIC, preterm premature rupture of membranes (PPROM), PTL, placental abruption, neonatal outcomes such as birth weight, Apgar score at 1 min and 5 min, stillbirth or immediate death rate, overall perinatal survival rate, neonatal intensive care unit (NICU) admission rate, and composite complications including respiratory distress syndrome, intraventricular hemorrhage, necrotizing enterocolitis, cardiovascular disease, or sepsis. Perioperative clinical details included GA at the time of the PEIC, preoperative cervical dilatation, and prolapse of the membranes into the vagina. Perioperative laboratory and sonographic findings included various immune or inflammatory indices such as white blood cell, C-reactive proteins, neutrophil to lymphocyte ratio (NLR), or platelet to lymphocyte ratio, biomarkers such as fetal fibronectin (fFN) or insulin-like growth factor binding protein-1 (IGFBP-1) (ActimPROM, Actim Oy, Espoo, Finland), vaginal microbiome such as mycoplasma or ureaplasma, and postoperative CL or funneling on transvaginal ultrasonography.

Additionally, we compared the demographic characteristics and perioperative clinical, laboratory, and sonographic findings according to the delivery status at GA 28 weeks in all patients who underwent PEIC, irrespective of singleton and twin pregnancies. In addition, various risk factors were analyzed to predict PTB before GA 28 weeks.

Statistical analyses were performed using the Statistical Package for Social Science (SPSS) version 26.0 (SPSS Inc., Chicago, IL, USA). Categorical variables were analyzed using chi-squared and Fisher exact tests when the sample size was five or less. Continuous variables were compared using the Mann–Whitney *U* test or Student *t*-test according to the distribution. Receiver operating characteristic (ROC) curves were generated to identify the optimal cutoff value of the predictors. The area under the ROC curve (AUC) was measured for each predictor. Predictive factors associated with PTB before GA 28 weeks were identified in the bivariate analysis. A multivariate logistic regression, with results presented as aOR with 95% CI, was performed to identify risk factors associated with PTB before GA 28 weeks following PEIC. A *p* < 0.05 was considered statistically significant.

## 3. Results

A total of 147 patients, including 28 and 119 with twin and singleton pregnancies, respectively, who underwent PEIC were identified. All cases of PEIC were successfully treated without intraoperative iatrogenic rupture of the membranes. Table 1 compares the demographic characteristics of twin and singleton pregnancies. Women with twin pregnancies had a higher rate of ART, whereas the rates of multiparity were significantly higher in women with singleton pregnancies. Other demographic characteristics did not differ between the two groups.

Table 2 shows twin and singleton pregnancies’ perioperative clinical, laboratory, and sonographic findings. Notably, the two groups had no differences in the GA at the PEIC, preoperative cervical dilatation, or prolapsed membranes into the vagina. The range and mean of preoperative cervical dilatation were as follows: twin, 1–5.8 cm with a mean of 2.91 cm; and singleton, 1–5.9 cm with a mean of 2.87 cm (*p* = 0.865). The average GA at PEIC in twin gestations was 21.3 weeks (range, 17.2–24.6 weeks), and that in singleton gestations was 21.1 weeks (range, 15.6–24.4 weeks) (*p* = 0.476). The other perioperative details were not significantly different between the two groups.

Table 3 compares pregnancy and neonatal outcomes between twin and singleton pregnancies. The primary outcome, the PTB rate before 28 weeks, was not different between the two groups (twin, 53.6% vs. singleton, 36.1%; *p* = 0.131; odd ratio, 0.490; and 95% confidence interval, 0.213–1.126). Similarly, GA at delivery (twin, 28.87 weeks; interquartile range [IQR]: 20.8–37.0 weeks vs. singleton, 30.67 weeks; IQR: 16.5–40.57 weeks, *p* = 0.148), the rate of PTB before 32 weeks and 34 weeks, PEIC-to-delivery latency, PTL, placenta abruption and neonatal outcomes except birth weight were not statistically different. However, the rate of term delivery, defined as delivery at GA ≥ 37 weeks (twin, 3.6% vs. singleton, 21.8%, *p* = 0.028) and neonatal birth weight (twin, 1314g; IQR: 320–3130 vs. singleton, 1807 g; IQR: 108–4520 g, *p* = 0.001) were lower in twin pregnancies than in single pregnancies, and the rate of cesarean section (twin, 85.7% vs. singleton, 47.1%, *p* < 0.001) and PPROM (twin, 39.3% vs. singleton, 19.3%, *p* = 0.043) were significantly longer and higher in twin pregnancies than in singleton pregnancies.

As there were no statistical differences in PTB before GA 28 weeks, and in most pregnancy and neonatal outcomes between twin and singleton pregnancies, we further analyzed the whole population according to delivery at GA 28 weeks regardless of twin or singleton pregnancies. Table 4 compares the demographic characteristics between the two groups based on the GA at delivery before or after 28 weeks. A total of 58 women delivered at GA < 28 weeks (group A), and 89 delivered at GA ≥ 28 weeks (group B). The rate of twin pregnancies was not different (group A, 25.9% vs. group B, 14.6%; *p* = 0.131), and other demographic characteristics were not different between the two groups (Table 4).

On preoperative physical examination, group A was more likely to have advanced cervical dilatation, bulging membranes into the vagina than group B. Group A showed a mean of 3.28 cm cervical dilatation before PEIC, whereas group B showed a mean of 2.61 cm cervical dilatation (*p* < 0.002); group A demonstrated more cases of preoperative prolapse membranes into the vagina (group A, 81.0% vs. group B, 64.0%, *p* = 0.041) (Table 5). Regarding preoperative laboratory findings, group A showed a higher level of CRP (group A, 11.57 mg/L vs. group B, 8.03 mg/L, *p* = 0.028), NLR (group A, 8.84 vs. group B, 7.03, *p* = 0.035), and a higher incidence of positive fFN or IGFBP-1 than group B (group A, 68.1% vs. group B, 46.7%, *p* = 0.043). Moreover, group A demonstrated a shorter postoperative CL than group B (group A, 24.74 mm vs. group B, 27.16 mm, *p* = 0.049). However, other perioperative clinical, laboratory, and sonographic findings did not differ between the two groups (Table 5).

As expected, the pregnancy and neonatal outcomes differed between groups A and B. Patients in group A had poorer pregnancy and neonatal outcomes than those in group B (Table 6). The rate of PTL was significantly higher in group A, whereas the rates of PPROM and placental abruption were not different between the two groups. In addition, the mean birth weight and the rate of final viable pregnancy were lower in group A than in group B, and the rates of NICU admission, stillbirth or immediate death, and composite neonatal complications were higher in group A than in group B (Table 6).

**Figure 1:**
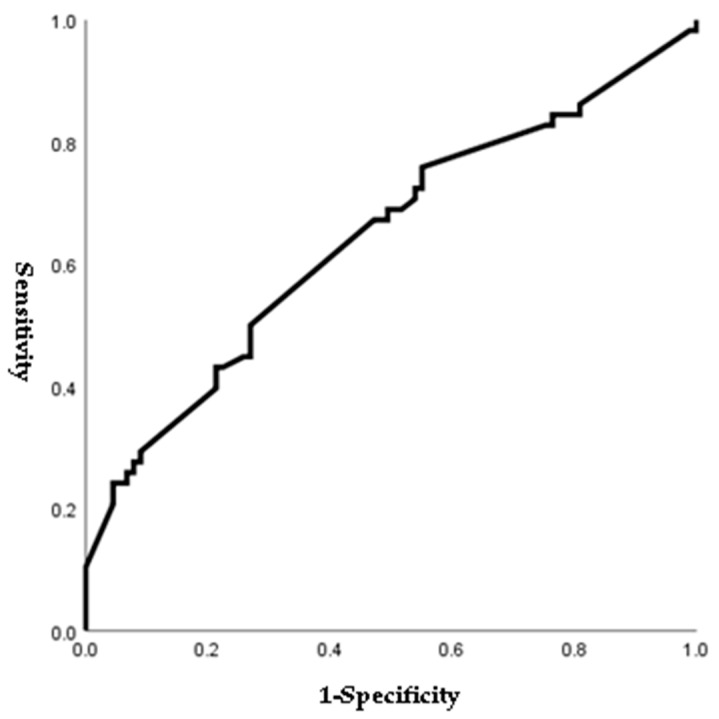
ROC curve of preoperative cervical dilatation to predict preterm birth before 28 weeks of gestations. (AUC, 0.643; cutoff, 3.0 cm; Sn, 67.2%; Sp, 52.8%, *p*-value = 0.003).

**Figure 2:**
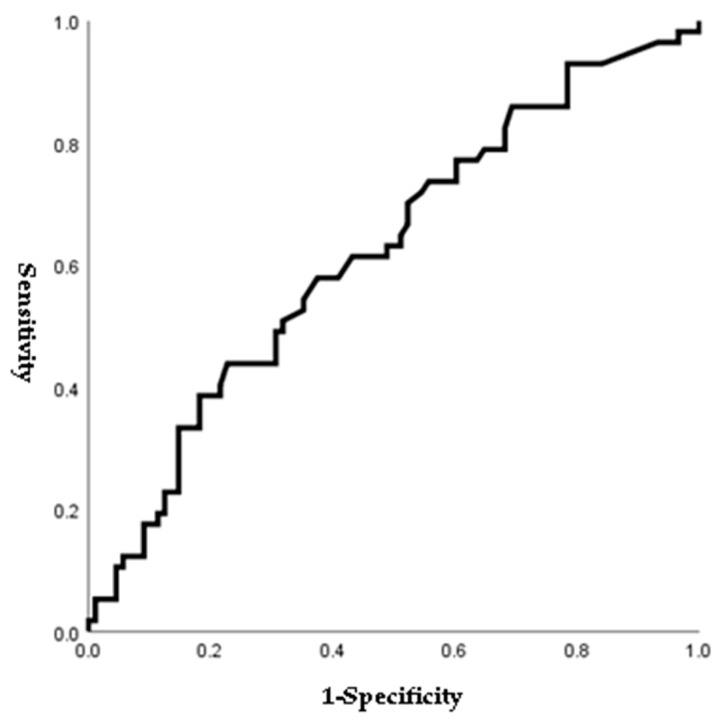
ROC curve of preoperative CRP to predict preterm birth before 28 weeks of gestations. (AUC, 0.622; cutoff, 11.1 mg/L; Sn, 43.9%; Sp, 77.3%, *p*-value = 0.048).

**Figure 3:**
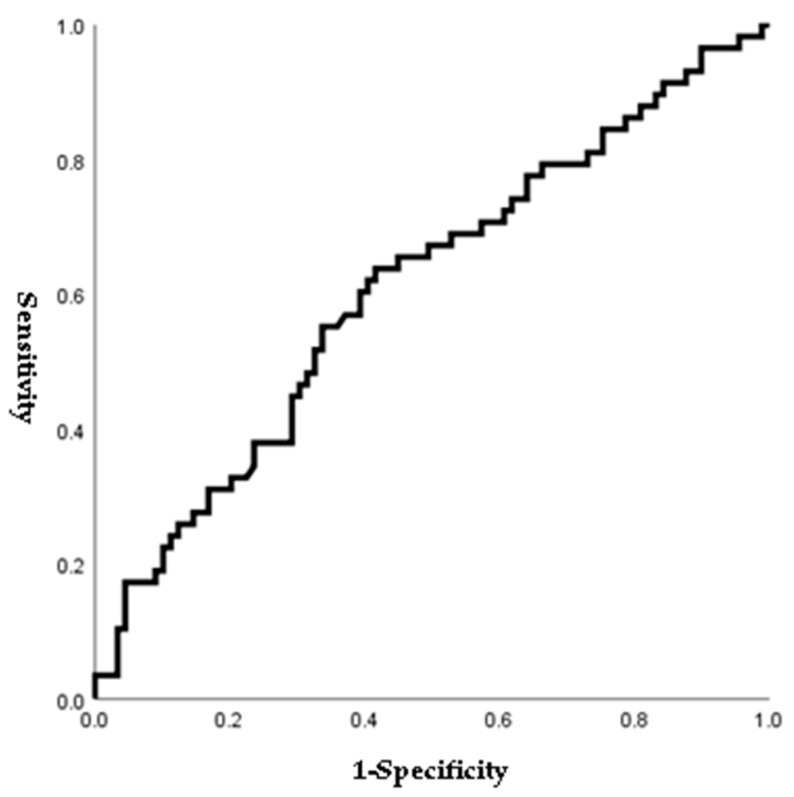
ROC curve of preoperative NLR to predict preterm birth before 28 weeks of gestations. (AUC, 0.609; cutoff, 7.1; Sn, 54.4%; Sp, 65.9%, *p*-value = 0.025).

**Figure 4:**
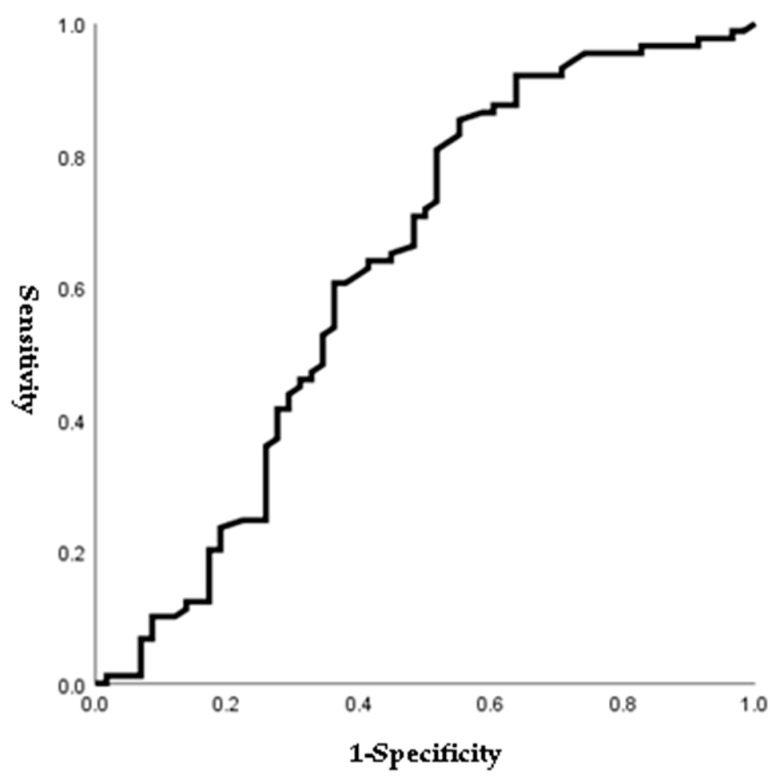
ROC curve of postoperative cervical length to predict preterm birth before 28 weeks of gestations. (AUC, 0.629; cutoff, 21.6 mm; Sn, 54.4%; Sp, 65.9%, *p*-value = 0.008).

Figure 1, Figure 2, Figure 3 and Figure 4 outline the ROC curve for PTB at GA < 28 weeks based on preoperative cervical dilatation, CRP, NLR, and postoperative CL. The AUCs for the preoperative cervical dilatation and postoperative CL were 0.643, 0.622, 0.609, and 0.629, respectively. The cutoff values for predicting PTB before GA 28 weeks were 3.0 cm for preoperative cervical dilatation, 11.1 mg/L for CRP, 7.1 for NLR, and 21.6 mm for postoperative CL.

Univariable analysis for predicting PTB before GA 28 weeks is demonstrated in Table 7. Preoperative cervical dilatation ≥ 3.0 cm, prolapsed membranes into the vagina, preoperative CRP ≥ 11.1, and NLR ≥ 7.1 were associated with increased odds of PTB before GA 28 weeks. In addition, positive fFN or IGFBP-1 levels were associated with an increased risk of PTB before GA 28 weeks. Furthermore, postoperative CL < 21.6 mm was significantly associated with increased odds of PTB before GA 28 weeks.

We further performed multivariate analysis to predict PTB before GA 28 weeks (Table 8). Interestingly, neither cervical dilatation ≥ 3.0 cm nor prolapsed membranes showed statistical significance in predicting PTB before GA 28 weeks. CRL ≥ 11.1 mg and NLR ≥ 7.1 also were not associated with preterm birth before GA 28 weeks. Only positive fFN or IGFBP-1 (aOR = 2.311, *p* = 0.039, 95% CI 1.042–5.125) and postoperative CL < 21.6 mm (aOR = 4.353, *p* = 0.001, 95% CI 1.820–10.411) were strongly associated with the prediction of PTB before GA 28 weeks following PEIC (Table 8).

## 4. Discussion

Our study demonstrated that the overall pregnancy outcomes after PEIC did not differ between twin and singleton pregnancies. There were no significant differences in the delivery rates at GA < 28 weeks. Although neonatal birth weight was smaller in twin pregnancies than in singletons, the GA at delivery, the incidence of delivery at GA < 34 weeks, 32 weeks, latency of PEIC to delivery, and other pregnancy and neonatal outcomes did not differ between the two groups. Moreover, we thoroughly analyzed multiple factors using perioperative clinical, laboratory, and sonographic findings through various statistical methods, finally suggesting postoperative short CL and positive fFN or IGFBP-1 as predictive factors for PTB before 28 weeks of gestations following PEIC.

According to the US National Vital Statistics, in 2015, more than 50% of twins were delivered preterm, and preterm delivery occurred more frequently in twin pregnancies than in singleton pregnancies [11]. Our study also showed that the incidence of term delivery in twin gestations was lower than in singletons (3.6% vs. 21.8%, *p* = 0.028). In addition, the incidence of premature rupture of membranes during pregnancy was higher in twin gestations than in singletons. Since multiple pregnancies tend to be more affected by uterine distention and stretch, there was a difference between the two groups. Nevertheless, the mean GA at delivery, incidence of preterm delivery at <34 weeks, 32 weeks, and 28 weeks, and other pregnancy outcomes did not differ between the two groups. These findings highlight that PEIC in twin gestations improves pregnancy outcomes similar to those in singleton pregnancies.

We reported that the mean neonatal birth weight was lower in twin pregnancies than in singletons (1314 g vs. 1807 g, *p* = 0.001). This difference was due to the lower incidence of term delivery in twin gestations than in singletons. However, the rates of neonatal survival, NICU admission, and composite neonatal complications did not differ between groups. This emphasizes the finding that PEIC in twin pregnancies improves overall neonatal outcomes compared with singletons.

In our study, the primary outcome was preterm delivery before GA 28 weeks. Various studies have revealed that GA at delivery affects neonatal survival rate and composite outcomes, and the neonatal survival rate was over 90% after GA 28 weeks [1,2,4]. According to a 2016 contemporary cohort study, the neonatal overall survival rate was approximately 96%, and the neonatal survival rate without severe morbidities was approximately 53%; this was why we selected PTB at GA 28 weeks as the primary outcome [2].

However, the effects of PEIC on twin pregnancies remain controversial. According to a 2015 meta-analysis, cerclage in twin pregnancies did not decrease the rate of PTB at GA < 34 weeks and did not improve perinatal outcomes compared with control women who did not receive cerclage [12]. Moreover, a previous Cochrane meta-analysis showed that cerclage placement was not associated with benefits but with worse neonatal outcomes [13].

However, recent studies reported successful outcomes of PEIC in twin gestations like our study [14,15,16,17,18]. A recent randomized controlled trial (RCT) concluded that PEIC in twin gestations was associated with a 50% decrease in early PTB under GA 28 weeks and a 78% decrease in perinatal mortality [15]. As this RCT included PEIC with antibiotics and indomethacin, we could not conclude whether the decreased PTB and perinatal mortality were solely due to PEIC. In addition, a recent meta-analysis reported that cerclage was associated with a significant reduction in PTB in twin pregnancies with a CL of <15 mm or a dilated cervix of >10 mm [7,14,18]. Compared to our study, this meta-analysis included populations with short CLs. This population heterogeneity might have caused the slight differences observed in our study.

Although a few previous studies have suggested the promising effects of PEIC in twin pregnancies, limited data are available on identifying predictive factors, as in our study. Most previous studies have only compared and analyzed the clinical outcomes of PEIC in twin gestations [6,14,18,19]. We thoroughly analyzed various perioperative clinical, laboratory, and sonographic findings and performed various statistical trials to identify perioperative risk factors for predicting PTB before 28 weeks of gestation following PEIC in singleton and twin pregnancies. Through various statistical methods, we found that postoperative CL < 21.6 mm and positive fFN or IGFBP-1 levels were risk factors for PTB before 28 weeks. This finding is consistent with those of Miller et al. [19]. They demonstrated that PEIC in twin pregnancies might have obstetric outcomes similar to those of singletons, and a digital CL of <2 cm appeared to be a risk factor, particularly in women with twin gestations. However, this study analyzed only perioperative clinical findings associated with PTB before GA 28 weeks, compared to our study, which included various perioperative clinical, laboratory, and sonographic findings. In addition, digital CL is a subjective value that different implementers could change compared to sonographic CL, which is an objective value.

The clinical utility of fFN and IGFBP-1 as predictors of PTB has been previously studied [20,21,22,23,24]. Melchor et al. reported that pooled positive predictive values were 34.1% (95% CI: 29–39%) for fFN and 35.2% (95% CI: 31–40%) for IGFBP-1, and pooled negative predictive values were 93.3% (95% CI: 92–99%) for fFN and 98.7% (95% CI: 98–99%) for IGFBP-1 [19]. Therefore, the American College of Obstetricians and Gynecologists does not recommend screening for fFN to predict or detect PTB [23]. However, the results of only a few studies are consistent with our study. Tekesin et al. demonstrated that fFN was more effective in predicting the risk of delivery within 7 days in symptomatic patients, and one prospective observational study reported that IGFBP-1 was a more reliable test than fFN in predicting preterm delivery [19]. In addition, Jun et al. demonstrated that positive fFN and CL could be used to manage women with symptomatic PTL because they could identify women at risk of delivery before 37 weeks of gestation [24].

Our study analyzed various inflammatory indices and considered their possible predictive factors. Since a few previous studies have reported that NLR is associated with PTB or premature rupture of membranes before labor, we performed the ROC curve and logistic regression analyses to prove the predictive power of NLR [25,26]. The ROC curve for NLR is shown in Figure 3, and the cutoff value for NLR was 7.1 (AUC: 0.609; sensitivity: 54.4%; specificity: 65.9%). Patients who delivered before GA 28 weeks showed a higher rate of NLR > 7.1 than those who delivered after GA 28 weeks. In addition, NLR > 7.1 was associated with PTB before 28 weeks of gestations in bivariable analysis (Table 7). However, multivariate analysis showed no difference (OR: 2.05; 95% CI: 0.94–4.49; *p* = 0.073) (Table 8).

To the best of our knowledge, our study is the first single-center study to analyze risk factors using perioperative clinical, laboratory, and sonographic findings to predict PTB before 28 weeks of gestation through multiple statistical trials and suggest the importance of postoperative CL following PEIC and positive fFN or IGFBP-1 as risk factors for PTB. However, our study has some limitations. First, this was a retrospective study performed at a single center. Therefore, unmeasured confounding variables may still exist, although we attempted to adjust for confounders. Second, we performed a sonographic examination of the CL and funneling on postoperative day 1 and did not include a serial check of the CL following PEIC owing to dynamic changes in serial CL.

However, this study has several limitations. First, selection bias was possible due to the retrospective nature of the study. Second, we designated singleton pregnancies as a control group instead of twin pregnancies who had not undergone PEIC. This is an indirect comparison to prove the effectiveness of PEIC in twin pregnancies. So, evidence is not enough.

To overcome the aforementioned limitations, a multicenter, randomized trial is required to assess the efficacy of PEIC in twin gestations. In addition, prospective studies on biomarkers in the amniotic fluid, serum, or placenta and serial CL are recommended to determine the underlying mechanisms of PEIC and other predictive values to improve pregnancy and neonatal outcomes.

## 5. Conclusions

PEIC in twin pregnancies showed favorable perinatal outcomes comparable to those in singleton pregnancies. There were no differences in the rate of PTB at GA < 28 weeks, mean GA at delivery, or neonatal outcomes between singleton and twin pregnancies. Moreover, positive fFN or IGFBP-1 and postoperative CL < 21.6 mm might be risk factors for PTB at GA < 28 weeks following PEIC. However, evidence is not enough.

## Figures and Tables

**Table 1:** The comparison of demographic characteristics between twins and singleton underwent PEIC.

	Twin (n = 28)	Singleton (n = 119)	*p*-Value
Maternal age (years)	33.21 ± 3.25	34.08 ± 3.82	0.268
Elderly gravida ^a^	8 (28.6%)	59 (49.6%)	0.058
Parity			0.011 *
Primiparous	22 (78.6%)	61 (51.3%)	
Multiparous	6 (21.4%)	58 (48.7%)	
BMI (kg/m^2^)	26.74 ± 5.69	25.67 ± 4.56	0.287
BMI ≥ 30	8 (28.6%)	20 (16.8%)	0.182
Prior preterm birth or second-trimester loss	2 (7.1%)	12 (10.1%)	0.633
Prior cervix operation	0 (0%)	2 (1.7%)	0.490
Assisted reproductive technology	20 (71.4%)	21 (17.6%)	<0.001 *

Data are expressed as means (standard deviations) and numbers (percentages). *, which means statistical significance. ^a^, which means age over 35 years. PEIC, physical examination-indicated cerclage; BMI, body mass index.

**Table 2:** The comparison of perioperative clinical, laboratory and sonographic findings between twin and singleton underwent PEIC.

	Twin (n = 28)	Singleton (n = 119)	*p*-Value
GA at PEIC (weeks)	21.34 ± 1.74	21.05 ± 1.97	0.476
Cervical dilation (cm)	2.91 ± 1.35	2.87 ± 1.31	0.865
Prolapsed membrane into vagina	20 (71.4%)	84 (70.6%)	0.930
WBC	11,889.29 ± 3652.80	11,181.76 ± 3002.84	0.284
CRP (mg/L)	9.05 ± 6.30	9.51 ± 10.10	0.817
NLR	8.67 ± 5.86	7.53 ± 4.40	0.252
PLR	189.63 ± 68.07	190.46 ± 72.79	0.956
fFN/IGFBP-1	16 (57.1%)	62 (52.1%)	0.678
Mycoplasma	0 (0%)	3 (2.5%)	0.396
Ureaplasma	11 (39.3%)	53 (44.5%)	0.676
Postoperative CL	25.79 ± 5.69	26.30 ± 7.13	0.685
Postoperative funneling	7 (25.0%)	18 (15.1%)	0.262

Data are expressed as means (standard deviations) and numbers (percentages). PEIC, physical examination-indicated cerclage; GA, gestational age; WBC, white blood cell; CRP, c-reactive protein; NLR, neutrophil–lymphocyte ratio; PLR, platelet lymphocyte ratio; fFN, fetal fibronectin; IGFBP-1, insulin growth factor binding protein-1; CL, cervical length.

**Table 3:** The comparison of pregnancy and neonatal outcomes between twins and singleton underwent PEIC.

	Twin (n = 28)	Singleton (n = 119)	*p*-Value
GA at delivery (weeks)	28.87 ± 5.15	30.67 ± 6.03	0.148
<28 weeks	15 (53.6%)	43 (36.1%)	0.131
<32 weeks	19 (67.9%)	64 (53.8%)	0.208
<34 weeks	19 (67.9%)	78 (65.5%)	0.816
Term delivery (≥37 weeks)	1 (3.6%)	26 (21.8%)	0.028 *
PEIC-to-delivery latency (days)	52.75 ± 34.15	67.3 ± 42.30	0.059
Cesarean section	24 (85.7%)	56 (47.1%)	<0.001 *
PPROM	11 (39.3%)	23 (19.3%)	0.043 *
PTL	18 (64.3%)	68 (57.1%)	0.530
Placenta abruption	1 (3.6%)	4 (3.4%)	0.956
Birth weight (g)	1313.98 ± 824.65 ^a^	1806.60 ± 1097.55	0.001 *
Apgar score at 1 min	4.54 ± 3.00 ^a^	5.15 ± 3.11	0.219
Apgar score at 5 min	6.04 ± 3.21 ^a^	6.65 ± 3.09	0.229
Stillbirth/immediate death	9/56 (16.1%) ^a^	17 (14.3%)	0.821
Final Viable pregnancy	50/56 (89.3%) ^a^	110 (92.4%)	0.565
NICU admission	38/56 (67.9%) ^a^	80 (67.2%)	0.934
Composite complication	16/56 (28.6%) ^a^	31 (26.1%)	0.719

Data are expressed as means (standard deviations) and numbers (percentages). *, which means statistical significance. ^a^, which means variables of the total of 56 neonates, which included 28 twin pregnancies (56 neonates), PEIC, physical examination-indicated cerclage; PPROM, preterm premature rupture of membranes; PTL, preterm labor.

**Table 4:** The comparison of demographic characteristics between GA at delivery < 28 weeks and ≥28 weeks.

	<28 Weeks (n = 58)	≥28 Weeks (n = 89)	*p*-Value
Maternal age (years)	34.52 ± 3.38	33.53 ± 3.90	0.989
Elderly gravida ^a^	31 (53.4%)	36 (40.4%)	0.131
Parity			0.309
Primiparous	36 (62.1%)	47 (52.8%)	
Multiparous	22 (37.9%)	42 (47.2%)	
BMI (kg/m^2^)	26.47 ± 5.27	25.48 ± 4.44	0.223
BMI ≥ 30	13 (22.4%)	15 (16.9%)	0.401
Prior preterm birth or Second-trimester loss	4 (6.9%)	10 (11.2%)	0.567
Prior cervix operation	1 (1.7%)	1 (1.1%)	0.759
Assisted reproductive technology	18 (31.0%)	23 (25.8%)	0.573
Twin pregnancy	15 (25.9%)	13 (14.6%)	0.131

Data are expressed as means (standard deviations) and numbers (percentages). ^a^, which means age over 35 years. PEIC, physical examination-indicated cerclage; BMI, body mass index.

**Table 5:** The comparison of perioperative clinical, laboratory, and sonographic findings between GA at delivery < 28 weeks and ≥28 weeks.

	<28 Weeks (n = 58)	≥28 Weeks (n = 89)	*p*-Value
GA at PEIC (weeks)	20.85 ± 1.99	21.27 ± 1.88	0.197
Cervical dilation (cm)	3.28 ± 1.43	2.61 ± 1.16	0.002 *
Cervical dilation ≥ 3.0 cm	39 (67.2%)	42 (47.2%)	0.019 *
Prolapsed membrane into vagina	47 (81.0%)	57 (64.0%)	0.041 *
WBC	11,517.76 ± 3287.59	11,185.39 ± 3044.50	0.532
CRP (mg/L)	11.57 ± 10.71	8.03 ± 8.33	0.028 *
CRP ≥ 11.1 mg/L	25 (43.9%)	21 (23.9%)	0.017 *
NLR	8.84 ± 5.66	7.03 ± 3.85	0.035 *
NLR ≥ 7.1	31 (53.4%)	30 (33.7%)	0.026 *
PLR	202.86 ± 73.98	182.13 ± 69.36	0.087
fFN/IGFBP-1	37 (63.8%)	41 (46.1%)	0.043 *
Mycoplasma	2 (3.4%)	1 (1.1%)	0.562
Ureaplasma	27 (46.6%)	37 (4163%)	0.611
Postoperative CL	24.74 ± 7.87	27.16 ± 5.98	0.049 *
Postoperative CL < 21.6 mm	26 (44.8%)	13 (14.6%)	<0.001 *
Postoperative funneling	13 (22.4%)	12 (13.5%)	0.182

Data are expressed as means (standard deviations) and numbers (percentages). *, which means statistical significance. PEIC, physical examination-indicated cerclage; GA, gestational age; WBC, white blood cell; CRP, c-reactive protein; NLR, neutrophil–lymphocyte ratio; PLR, platelet–lymphocyte ratio; fFN, fetal fibronectin; IGFBP-1, insulin growth factor binding protein-1; CL, cervical length.

**Table 6:** The comparison of pregnancy and neonatal outcomes between twin and singleton underwent PEIC.

	<28 Weeks (n = 58)	≥28 Weeks (n = 89)	*p*-Value
GA at delivery (weeks)	24.08 ± 2.15	34.40 ± 3.49	<0.001 *
PEIC-to-delivery latency (days)	22.59 ± 14.14	91.87 ± 27.69	<0.001 *
Cesarean section	36 (62.1%)	44 (49.4%)	0.175
PPROM	16 (27.6%)	18 (20.2%)	0.322
PTL	46 (79.3%)	40 (44.9%)	<0.001 *
Placenta abruption	4 (6.9%)	1 (1.1%)	0.079
Birth weight (g)	668.42 ± 223.45 ^a^	2350.72 ± 800.85 ^b^	<0.001 *
Apgar score at 1 min	1.97 ± 1.69 ^a^	7.09 ± 1.81 ^b^	<0.001 *
Apgar score at 5 min	3.47 ± 2.43 ^a^	8.59 ± 1.23 ^b^	<0.001 *
Stillbirth/immediate death	21/73 (28.8%) ^a^	5/102 (4.9%) ^b^	<0.001 *
Final Viable pregnancy	59/73 (80.8%) ^a^	101/102 (99.0%) ^b^	<0.001 *
NICU admission	58/73 (79.5%) ^a^	60/102 (58.8%) ^b^	0.005 *
Composite complication	27/73 (37.0%) ^a^	20/102 (19.6%)	0.015 *

Data are expressed as means (standard deviations) and numbers (percentages). *, which means statistical significance. ^a^, which means variables of a total of 73 neonates, which included 15 twin (30 neonates) and 43 singleton pregnancies (43 neonates). ^b^, which means variables of a total of 102 neonates, which included 13 twins (26 neonates) and 76 singleton pregnancies (76 neonates). PEIC, physical examination-indicated cerclage; PPROM, preterm premature rupture of membranes; PTL, preterm labor.

**Table 7:** Univariable analysis of factors associated with preterm delivery < 28 weeks of gestation.

	OR	95% CI	*p*-Value
Cervical dilation ≥ 3.0 cm	2.297	1.154–4.572	0.018 *
Prolapsed membrane	2.399	1.093–5.266	0.029 *
CRP ≥ 11.1 mg/L	2.493	1.217–5.105	0.013 *
NLR ≥ 7.1	2.258	1.147–4.447	0.019 *
fFN/IGFBP-1	2.063	1.046–4.066	0.037 *
Postoperative CL < 21.6 mm	4.750	2.170–10.398	<0.001 *

*, which means statistical significance. OR, odd ratio; CI, confidence interval; CRP, c-reactive protein; NLR, neutrophil–lymphocyte ratio; fFN, fetal fibronectin; IGFBP-1, insulin growth factor binding protein 1; CL, cervical length.

**Table 8:** Multivariable analysis of factors associated with preterm delivery < 28 weeks of gestation.

	aOR	95% CI	*p*-Value
Cervical dilation ≥ 3.0 cm	0.709	0.228–2.207	0.552
Prolapsed membrane	2.876	0.805–10.280	0.104
CRP ≥ 11.1 mg/L	1.606	0.712–3.623	0.254
NLR ≥ 7.1	2.049	0.935–4.490	0.073
fFN/IGFBP-1	2.311	1.042–5.125	0.039 *
Postoperative CL < 21.6 mm	4.353	1.820–10.411	0.001 *

*, which means statistical significance. aOR, adjusted odd ratio; CI, confidence interval; fFN, fetal fibronectin; IGFBP-1, insulin growth factor binding protein 1; CL, cervical length.

## Data Availability

The data and materials in this study are available from the corresponding author upon request.
